# Lidocaine Sensitizes the Cytotoxicity of Cisplatin in Breast Cancer Cells via Up-Regulation of *RAR*β2 and *RASSF1A* Demethylation

**DOI:** 10.3390/ijms151223519

**Published:** 2014-12-17

**Authors:** Kehan Li, Jianxue Yang, Xuechang Han

**Affiliations:** 1 Department of Anesthesiology, the First Affiliated Hospital of Henan Science and Technology University, Luoyang 471003, China; E-Mail: hanxuech@163.com; 2 Department of Neurology, the First Affiliated Hospital of Henan Science and Technology University, Luoyang 471003, China; E-Mail: jianxyang276@163.com

**Keywords:** lidocaine, DNA demethylation, cisplatin, breast cancer cells, apoptosis

## Abstract

It has been reported that lidocaine is toxic to various types of cells. And a recent study has confirmed that lidocaine exerts a demethylation effect and regulates the proliferation of human breast cancer cell lines. To recognize a potential anti-tumor effect of lidocaine, we evaluated the DNA demethylation by lidocaine in human breast cancer lines, MCF-7 and MDA-MB-231 cells, and determined the influence of demethylation on the toxicity to these cells of cisplatin, which is a commonly utilized anti-tumor agent for breast cancer. Results demonstrated that lidocaine promoted a significant global genomic demethylation, and particularly in the promoters of tumor suppressive genes (TSGs), *RAR*β*2* and *RASSF1A*. Further, the lidocaine treatment increased cisplatin-induced apoptosis and enhanced cisplatin-induced cytotoxicity. The combined treatment with both lidocaine and cisplatin promoted a significantly higher level of MCF-7 cell apoptosis than singular lidocaine or cisplatin treatment. Moreover, the abrogation of *RAR*β*2* or *RASSF1A* expression inhibited such apoptosis. In conclusion, the present study confirms the demethylation effect of lidocaine in breast cancer cells, and found that the demethylation of *RAR*β*2* and *RASSF1A* sensitized the cytotoxicity of cisplatin in breast cancer cells.

## 1. Introduction

Lidocaine is an aminoamide-type anesthetic, which is commonly used for regional anesthesia and pain relief, due to its rapid onset of action and intermediate efficacy. However, lidocaine occasionally causes neural injury in patients who have received spinal anesthesia using this agent [1,2]. And several studies indicate that lidocaine not only induces neurotoxicity with morphological changes such as cell axon collapse and cell swelling [3,4], but also promotes apoptosis through the mitochondrial pathway [5,6,7,8,9], and even induces necrosis [10,11,12]. Besides the mitochondrial dysfunction, increased intracellular Ca^2+^, Na^+^, and pH at least partly contribute to the lidocaine-induced cell apoptosis or necrosis [13,14], such as the toxicity of lidocaine to articular chondrocytes [15,16]. However, more detailed molecular mechanisms underlying this cytotoxicity of lidocaine have been little understood.

Studies have indicated that the silencing of tumor suppressor genes (TSGs) through methylation of their promoters is one of the causes of tumor development [17]. As one of the natural covalent modifications of chromatin, defined as epigenomic [18], the silencing of TSGs by DNA methylation induces mechanisms responsible for promoting tumor development such as uncontrolled cell growth, metastasis, and avoidance of apoptosis or maintaining angiogenesis. As an example, methylation of promoter regions of TSGs, such as *CDKN2A*, *VHL* and *BRCA1* render them inactive in cancer cells [17,19]. Moreover, DNA methylation may facilitate the mutation of TSGs. In more than 50% of solid tumors, the tumor suppressive *p53* gene is mutated, and 25% oft he mutations result from methylated cytosine to thymine in the CpG dinucleotides of this gene [20]. Subsequently, the quantitative analysis of DNA methylation profiles for more cancer-related genes also indicates a strong association of the TSG hypermethylation with colorectal cancers [21] or lung cancers [22].

Several anesthetics have been confirmed to exert demethylation effects and regulate the proliferation of human cancer cells. Procaine promotes DNA demethylation and inhibits the growth of the human breast cancer cell line, MCF-7 [23] and in human hepatoma cells [24]. Recently, lidocaine has also been recognized to promote DNA demethylation in a time- and dose-dependent manner in breast cancer cell lines, BT-20 and MCF-7* in vitro* [25]. And this agent has been confirmed to induce endoplasmic reticulum stress-associated apoptosis in a rat pheochromocytoma PC12 cell line [26]. However, it is not clear whether the demethylation effect of lidocaine exerts an anti-tumor effect, and it is not clear whether this anesthetic cooperates with other well-recognized anti-tumor agents, such as cisplatin in the anti-tumor process.

In the present study, we evaluated DNA demethylation by lidocaine in the human breast cancer cell lines MCF-7 and MDA-MB-231, and determined the cooperation of the demethylation-inducing agent with the toxicity of cisplatin, a commonly utilized anti-tumor agent for breast cancer.

## 2. Results

### 2.1. Lidocaine Promotes Global Genomic Demethylation of the CpG Island in Human Breast Cancer Lines

**Figure 1:**
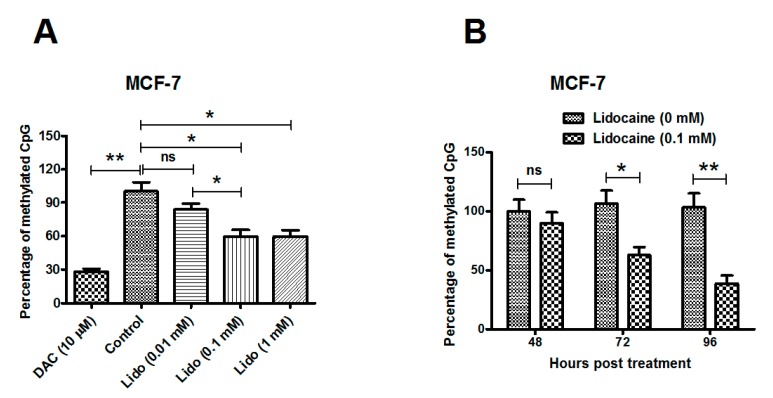

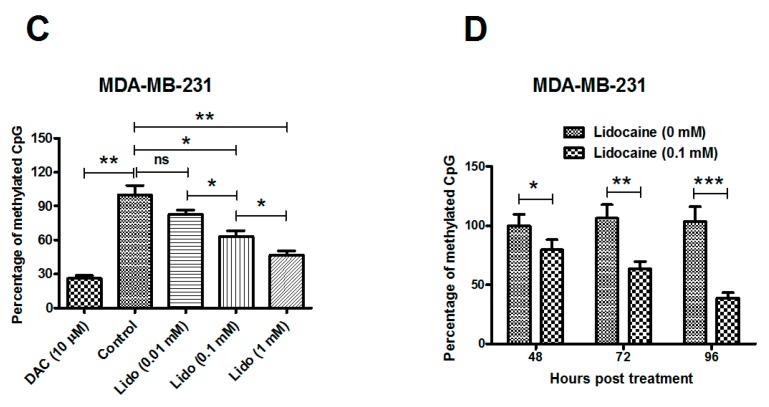
Global genomic demethylation of CpG islands promoted by lidocaine. (**A**) DNA methylation levels in MCF-7 breast cancer cells treated for 72 h with 0.01, 0.1 or 1 mM lidocaine treatment, or with 10 μM DAC treatment, respectively; (**B**) DNA methylation levels in MCF-7 cells treated with or without 0.1 mM lidocaine, for 48, 72 or 96 h; (**C**,**D**) DNA methylation levels in MDA-MB-231 breast cancer cells post above-mentioned treatment respectively. Results were expressed as mean ± SD for three independent experiments. Statistical significance was assessed by the unpaired Student’s *t*-test (* *p* ˂ 0.05, ** *p* ˂ 0.01, or *** *p* ˂ 0.001, ns: no significance).

Two human breast cancer lines, MCF-7 and MDA-MB-231 cells, were treated with various concentrations of lidocaine (0.01, 0.1 or 1 mM) for 72 h and the global DNA methylation of 5' CpG islands before and after each treatment was measured by sodium bisulfite DNA sequencing. As shown in Figure 1A (column 1 and 2), 10 μM 5-aza-2'-deoxycytidine (DAC, a demethylation agent as positive control) treatment significantly reduced the global methylation of the CpG island in MCF-7 cells, compared to the control MCF-7 cells (*p* ˂ 0.01). The lidocaine treatment with 0.1 or 1 mM also significantly promoted global genomic CpG island demethylation (Figure 1A, column 4 and 5; both *p* ˂ 0.05). To confirm the demethylation promotion by lidocaine, MCF-7 cells post 0.1 mM lidocaine for various hours were examined for global genomic methylation. Figure 1B demonstrates that the methylation level was significantly reduced by the 0.1 mM lidocaine treatment for either 72 or 96 h (*p* ˂ 0.05 for 72 h, and *p* ˂ 0.01 for 96 h), rather than at 48 h post treatment.

We then evaluated the global DNA methylation induced by lidocaine in MDA-MB-231 cells. Figure 1C indicates that the treatment with 0.1 or 1 mM lidocaine for 72 h also promoted global DNA demethylation (*p* ˂ 0.05 for 0.1 mM and *p* ˂ 0.01 for 1 mM), with a dose-dependence (either *p* ˂ 0.05 between the 0.01 and 0.1 mM treatment, or between the 0.01 and 0.1 mM treatment). Moreover, the promotion in MDA-MB-231 cells developed from 48 h post treatment, earlier than in MCF-7 cells (Figure 1D). The time-dependence of the promotion was also significant (*p* ˂ 0.01 or *p* ˂ 0.001), and the demethylation difference between the lidocaine and control groups increased with treatment time. Taken together, lidocaine promotes global genomic demethylation of the CpG islands in human breast cancer MCF-7 and MDA-MB-231 cells.

### 2.2. Lidocaine Ameliorates the Expression of RARβ2 and RASSF1A Genes in MCF-7 and MDA-MB-231 Cells

**Figure 2:**
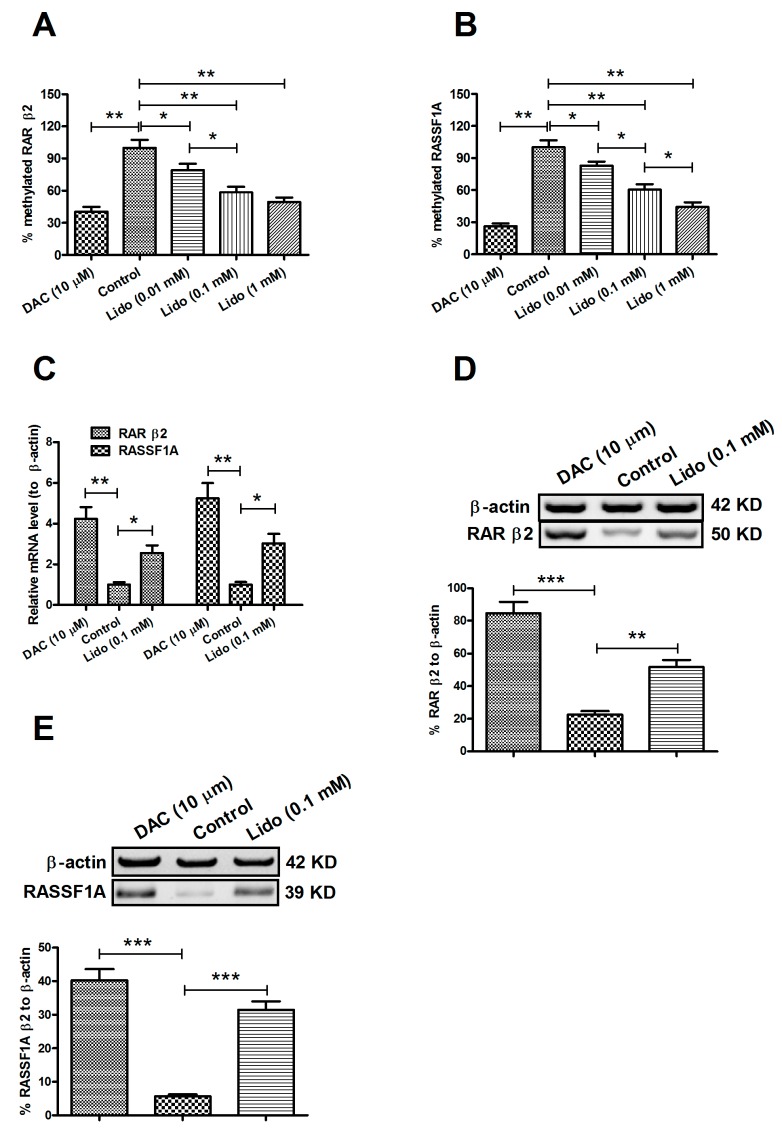
Lidocaine-promoted *RAR*β*2* and *RASSF1A* demethylation up-regulates the expression of both genes. Methylation levels of *RAR*β*2* (**A**) or *RASSF1A* (**B**) in MCF-7 cells treated for 72 h with 0.01, 0.1 or 1 mM lidocaine, or with 10 μM DAC, respectively; (**C**) mRNA level of *RAR*β*2* or *RASSF1A* in MCF-7 cells post treatment with 0.1 mM lidocaine or with 10 μM DAC for 72 h; Western blot assay for protein level of *RAR*β*2* (**D**) or *RASSF1A* (**E**) in MCF-7 cells with or without 0.1 mM lidocaine treatment for 96 h. Each value was expressed as mean ± SD for three independent tests. Statistical significance was assessed by the unpaired Student’s *t*-test (* *p* ˂ 0.05, ** *p* ˂ 0.01, or *** *p* ˂ 0.001).

The tumor suppressive genes, Retinoic acid receptor β (*RAR*β*2*) [27] and Ras Association Domain Family 1A (*RASSF1A*) [28,29] have been shown to be silenced by their promoter methylation in breast cancers. We evaluated the methylation levels of 5' CpG islands in these two TSG promoters, in MCF-7 cells with lidocaine treatment. Figure 2A,B indicates a similar methylation level in both genes to that of the global genomic CpG islands in MCF-7 cells; there was a significant dose-dependent demethylation by lidocaine of both *RAR*β*2* and *RASSF1A* genes (*p* ˂ 0.05 or *p* ˂ 0.01). To examine the influence of the promoter demethylation on the expression of *RAR*β2 and *RASSF1A*, we then determined the mRNA and protein levels of both molecules by real-time quantitative PCR (RT-qPCR) and western blot assay. As shown in Figure 2C, there was a significant promotion to *RAR*β*2* and *RASSF1A* mRNA levels either by 10 μM DAC (*p* ˂ 0.01 respectively), or by 0.1 mM lidocaine treatment for 72 h (*p* ˂ 0.05 respectively). Furthermore, the Western blot assay of both molecules reconfirmed the up-regulation of *RAR*β*2* and *RASSF1A* in protein level by the demethylation, which was promoted by 0.1 mM lidocaine treatment for 96 h (Figure 2D,E; *p* ˂ 0.01 or *p* ˂ 0.001). Therefore, the demethylation of *RAR*β*2* and *RASSF1A* promotes the expression of both genes.

### 2.3. Regulation of Lidocaine on the Viability of MCF-7 and MDA-MB-231 Cells

**Figure 3:**
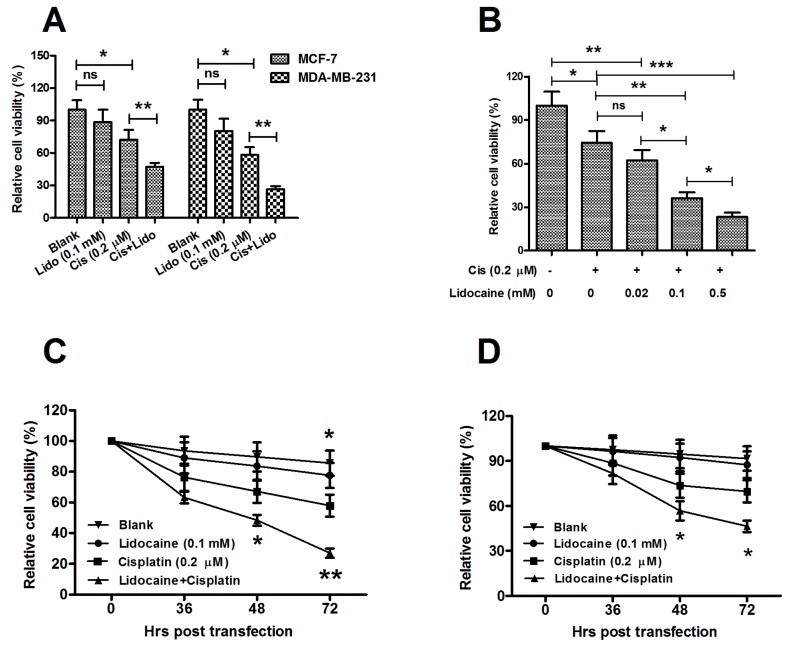
Lidocaine reduces the viability of MCF-7 and MDA-MB-231 cells in the presence of cisplatin. (**A**) Relative viability of MCF-7 and MDA-MB-231 cells treated with 0.2 μM cisplatin or (and) 0.1 mM lidocaine for 72 h; (**B**) Influence of 0.02, 0.1 or 0.5 mM lidocaine on the viability of 0.2 μM cisplatin-treated MCF-7 cells for 72 h; Relative viability of MCF-7 (**C**) or MDA-MB-231 (**D**) cells treated with 0.2 μM cisplatin or (and) 0.1 mM for 36, 48 or 72 h. All results were averaged for triple independent experiments. And each statistical significance was considered when *p* ˂ 0.05 or less. * *p* ˂ 0.05, ** *p* ˂ 0.01, or *** *p* ˂ 0.001, ns: no significance.

*RAR*β*2* has been recognized as a potent tumor suppressor. Expression of *RAR*β*2* in *RAR*β*2*-negative cancer cells restored retinoic acid-induced growth inhibition and caused decreased tumorigenicity [30]. Exogenous expression of *RAR*β*2* results both in RA-dependent and RA-independent breast cancer cell apoptosis [31,32]. The other TSG, *RASSF1A-*encoded protein was found to interact with such molecules as CNK1 [33], Nore1 [34], MDM2 [35], MAP1S [36] to induce cell death. Methylation of both TSGs has been reported to silence their expression and promote the tumorigenesis of breast cancers [27,28,29]. Given the significant demethylation effect of TSGs in breast cancer cells, we supposed a possible anti-tumor effect of lidocaine in the breast cancer cells. Firstly, we examined the viability of both MCF-7 and MDA-MB-231 cells post lidocaine treatment with a range of concentrations. Figure 3A (column 2 compared to column 1, or column 6 compared to column 5) shows that 0.1 mM lidocaine had no impact on the viability of both cell lines, 72 h post treatment. Also, either 0.01 or 1 mM lidocaine had an impact on cell viability (data not shown). Interestingly, the 0.1 mM lidocaine treatment decreased the cell viability reduction which was promoted by 0.2 μM cisplatin in both cell lines, 72 h post treatment (Figure 3A, column 4 or 8 compared to column 3 or 7; both *p* ˂ 0.01). To further determine the deterioration of cell viability induced by lidocaine, we re-evaluated the cell viability post lidocaine treatment, in the presence of 0.2 μM cisplatin. The cell viability reduction had been reconfirmed in the dosage of 0.1 or 0.5 rather than 0.02 mM (*p* ˂ 0.01 or *p* ˂ 0.001; Figure 3B). Figure 3C,D demonstrated that the cell viability reduction appeared significant from 48 h, and lasted to at least 72 h post treatment (*p* ˂ 0.05 or *p* ˂ 0.01 for MCF-7 cells and *p* ˂ 0.05 for MDA-MB-231 cells). Therefore, lidocaine reduced cell viability, in the presence of cisplatin, though its direct regulation was not observed in this study.

### 2.4. Lidocaine Enhances the Cytotoxicity of Cisplatin against MCF-7 Cells

**Figure 4:**
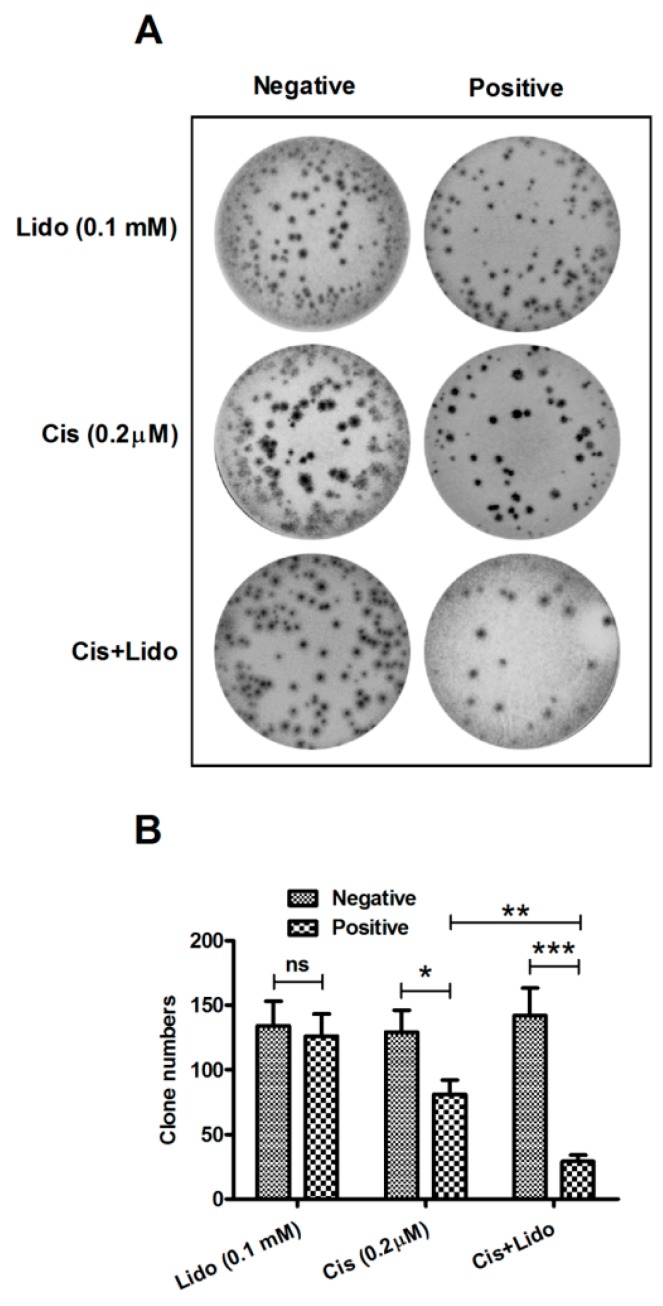
Colony formation of MCF-7 cells treated with lidocaine and/or cisplatin. (**A**) Colony formation of MCF-7 cells post the transfection with 0.2 μM cisplatin, 0.1 mM lidocaine or with both agents for 96 h; (**B**) The colony number in groups with or without the treatment of 0.2 μM cisplatin and/or 0.1 mM lidocaine. All experiments were performed independently in triplicate. Statistical significance was shown as * *p* ˂ 0.05, ** *p* ˂ 0.01, or *** *p* ˂ 0.001, ns: no significance.

Cisplatin has been clinically utilized for breast cancer treatment for decades [37,38], and its anti-proliferation effect has been confirmed to be abrogated [39] or sensitized [40] by other molecules. To investigate the regulation of lidocaine on the cisplatin-mediated anti-tumor effect, we evaluated the proliferation of MCF-7 cells, by clone assay, post treatment with 0.1 mM lidocaine, 0.2 μM cisplatin, or both agents. It was shown in Figure 4A that MCF-7 cells with or without the above-mentioned treatment formed clones after 96 h culture at 37 °C, and there was no obvious morphological difference of MCF-7 cells among these groups. Then the clone numbers in each group were calculated, and as shown in Figure 4B, the singular 0.1 mM lidocaine treatment did not significantly regulate clone forming (*p* > 0.05), whereas clone forming of MCF-7 cells was reduced in the presence of 0.2 μM cisplatin (*p* ˂ 0.001), and the reduction in clone number was more significant than the singular 0.2 μM cisplatin treatment (*p* ˂ 0.01). Taken together, lidocaine inhibits the MCF-7 cell proliferation in the presence of 0.2 μM cisplatin, and thus enhances the cytotoxicity of cisplatin against breast cancer cells.

### 2.5. Lidocaine Regulation of Apoptosis Promotion by Cisplatin in MCF-7 Cells

**Figure 5:**
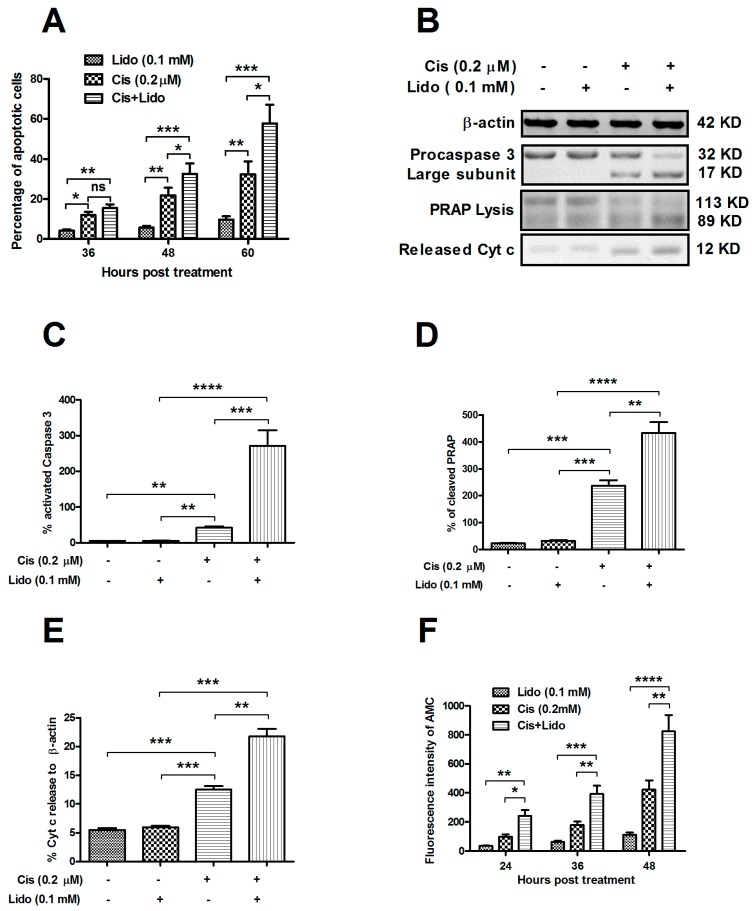
Lidocaine enhances cisplatin-induced apoptosis in MCF-7 cells. (**A**) Apoptotic cells induced by 0.2 μM cisplatin, or (and) 0.1 mM lidocaine for 36, 48 or 60 h; (**B**) Western blot analysis of activated caspase 3, cleaved PARP by activated caspase 3 and cytochrome c released from mitochondria in MCF-7 cells treated with 0.2 μM cisplatin, and/or 0.1 mM lidocaine for 60 h; (**C**–**E**) Relative levels of activated caspase 3, PARP cleavage and cytochrome c release to *β-actin* in MCF-7 cells treated with lidocaine, with cisplatin or with both agents; (**F**) Caspase 3 activity in lidocaine-, cisplatin- or both agents-treated MCF-7 cells, revealing by AMC Caspase Profiling Kit. Each value was averaged for three independent experiment results, and statistical significance was considered when *p* ˂ 0.05 or less, * *p* ˂ 0.05, ** *p* ˂ 0.01, *** *p* ˂ 0.001, **** *p* ˂ 0.0001, ns: no significance.

Cisplatin induces apoptosis in breast cancer cells [41,42], and the induction may be suppressed [43] or be sensitized [44]. To confirm whether cisplatin cytotoxicity enhancement by lidocaine is associated with cisplatin-induced apoptosis, we compared apoptosis-induction differences among singular treatment of cisplatin, of lidocaine and the combined treatment with both agents. Firstly, we measured the apoptosis level of MCF-7 cells, post treatment with 0.1 mM lidocaine, with 0.2 μM cisplatin, or with both agents, via flow cytometric analysis. It was shown that when subjected to 0.1 mM lidocaine for 36, 48 or 60 h, less than 10% MCF-7 cells underwent apoptosis, and there was no significant difference among them (Figure 5A; Figure S1). However, significantly more cells post treatment with 0.2 μM cisplatin underwent apoptosis (*p* < 0.05 for 36 h, *p* < 0.01 for 48 or 60 h; Figure 1A). Moreover, 0.1 mM lidocaine sensitized apoptosis promotion by cisplatin; the treatment with both agents promoted significantly higher levels of apoptosis in MCF-7 cells (*p* < 0.01 or *p* < 0.001 for 36, 48 or 60 h, compared to the lidocaine group; and *p* < 0.05 for 48 or 60 h, compared to the cisplatin group). Secondly, we analyzed by Western blot analysis apoptosis-associated molecules, such as procaspase 3, which is cleaved in apoptosis and performs as an apoptosis executor, PARPPARP, which is cleaved by activated caspase 3, and cytochrome c, which is released from mitochondria in apoptosis. Figure 5B–E demonstrates that singular treatment with 0.1 mM lidocaine had no significant regulation on procaspase 3 activation, PARP cleavage and cytochrome c release, all of which were promoted by 0.2 μM cisplatin. Moreover, the cisplatin-mediated promotion was sensitized by lidocaine; treatment with both agents promoted higher levels of the activation of above-mentioned molecules than singular cisplatin treatment (*p* < 0.01 or *p* < 0.001, compared to the cisplatin group). Finally, we examined the caspase 3 activity with the fluorophore 7-amino-4-methylcoumarin (AMC)-conjuncting kit. Figure 5F confirms the sensitization of lidocaine on the cisplatin-promoted caspase 3 activity; treatment with both agents promoted a significantly higher level of AMC fluorescence intensity than singular lidocaine or cisplatin treatment (*p* < 0.05 to *p* < 0.0001). Therefore, lidocaine sensitizes the apoptosis promotion by cisplatin in MCF-7 cells.

### 2.6. Abrogation of the RARβ2 and RASSF1A Genes Blocks the Apoptosis Promotion by Lidocaine and Cisplatin in MCF-7 Cells

**Figure 6:**
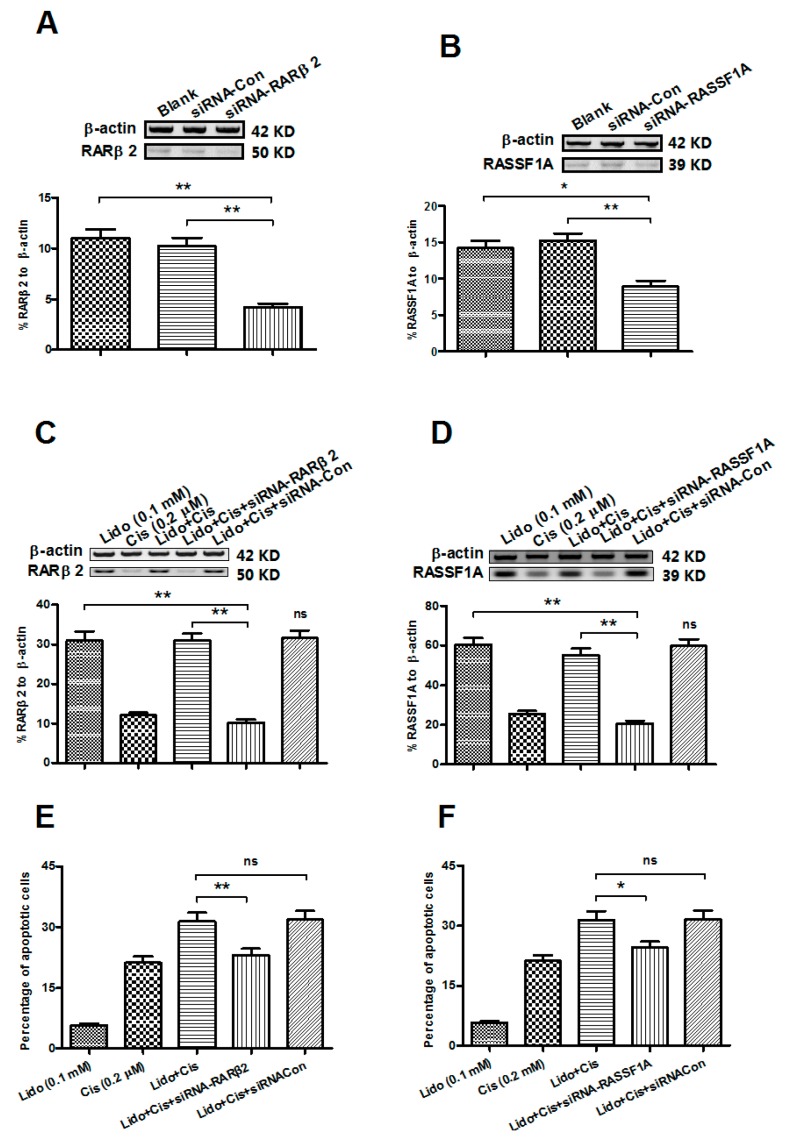
Transfection of *RAR*β*2-* or *RASSF1A-*specific siRNA inhibits the lidocaine and cisplatin-induced apoptosis in MCF-7 cells. (**A**–**D**) Western blot analysis of *RAR*β*2* (**A**,**C**) or RASSF1A (**B**,**D**) expression in MCF-7 cells post-transfection of the *RAR*β*2-* or *RASSF1A*-specific siRNA, without (**A**,**B**) or with (**C**,**D**) the lidocane and/or cisplatin treatment; (**E**,**F**) Apoptotic cells induced by the combined treatment with 0.1 mM lidocaine and 0.2 μM cisplatin for 48 h, post-transfection of *RAR*β*2-* (**E**) or *RASSF1A*-specific siRNA (**F**) * *p* ˂ 0.05, ** *p* ˂ 0.01, ns: no significance.

To further investigate the association of the lidocaine-promoted expression of *RAR*β*2* or *RASSF1A* with the MCF-7 cell apoptosis, we abrogated the expression of *RAR*β*2* or *RASSF1A* with the siRNA targeting either gene, and then determine the influence on the MCF-7 cell apoptosis, which was caused by the combined treatment with lidocaine and cisplatin. As shown in Figure 6A,B, the expression of *RAR*β*2* or *RASSF1A* was significantly down-regulated, 48 h post the siRNA–*RAR*β*2* or siRNA–*RASSF1A* transfection, in MCF-7 cells. Moreover, the donw-regulation of the *RAR*β*2* or *RASSF1A* expression was also confirmed in in the lidocaine- and cisplatin-treated MCF-7 cells, compared to the siRNA–control (Figure 6C,D; either *p* < 0.01). We then evaluated the influence of *RAR*β*2* or *RASSF1A* abrogation on the lidocaine- and cisplatin-induced apoptosis of MCF-7 cell cells. Figure 6E,F indicated that either siRNA–*RARβ2* or siRNA–*RASSF1A* transfection significantly inhibited the apoptosis of the MCF-7 cells, subject to 0.1 mM lidocaine and 0.2 μM cisplatin, for 48 h (*p* < 0.05 or *p* < 0.01). Therefore, the up-regulated expression of *RAR*β*2* or *RASSF1A* by the lidocaine-mediated demethylation of either gene contributed to the lidocaine- and cisplatin-induced apoptosis. In addition, to exclude a possibly different influence of siRNA transfection on cell apoptosis, we evaluated apoptosis induction of MCF-7 cells post-transfection with siRNA–*RASSF1A*, siRNA–*RAR*β*2*, or with siRNA control. No significant difference was found among the three groups (Figure S2).

## 3. Discussion

Studies have indicated that silencing of TSGs by promoter methylation contributes to tumor development [17]. TSG silencing by DNA methylation induces mechanisms such as uncontrolled cell growth, metastasis, reduced apoptosis or maintainence of angiogenesis, all of which promote tumor development [17,19]. Moreover, DNA methylation may facilitate the mutation of TSGs, such as mutated *p53* [20], or contribute directly to tumorigenesis, such as the hypermethylation in *APC-1A*, *CDKN2* and *RASSF1A* genes in colorectal cancer [21], or the hypermethylation in *MTHFR* gene in lung cancer [22]. On the other side, promotion of DNA demethylation decreases or inhibits the incidence or the development of cancers [45,46,47,48]; DNA demethylation agent 5-aza-2'-deoxycytidine decreases the incidence and inhibits the growth of gastric cancers [45,48]; miRNA-34b inhibits prostate cancer through demethylation, activating chromatin modifications [46]; and DNA demethylation by 5-aza-2-deoxycytidine abrogates 17 β-estradiol-induced cell growth of human breast cancer cells [49]. However, the mechanisms of the anti-tumor effects of DNA demethylation agents still need to be clarified.

Lidocaine has recently been indicated to promote DNA demethylation in breast cancer cell lines [25]. The present study has confirmed lidocaine promotion of global genomic demethylation of CpG islands in MCF-7 and MDA-MB-231 cells; treatment with 0.1 or 1 mM lidocaine for 72 h promoted global DNA methylation in both cell lines with time-dependent and dose-dependent repeatability. The retinoic acid receptor β (*RAR*β*2*) was shown to mediate the growth-inhibitory effect of retinoic acid by promoting apoptosis in human breast cancer cells [32] and to be silenced by promoter methylation in breast cancer [27].* RAR*β*2* over-expression restored retinoic acid-induced growth arrest and apoptosis in breast cancer cells [50]. Ras Association Domain Family 1A (*RASSF1A*), a putative tumor suppressor gene from the 3p21.3 locus, is another well-recognized tumor suppressive gene in various types of tumors [51,52,53]. The hypermethylation of CpG Islands in *RASSF1A* occurs in a large percentage of human breast cancers [54]. Results here indicate that hypermethylation in both genes was reduced significantly by lidocaine treatment, and that the demethylation induced by lidocaine ameliorates the reduced expression of *RAR*β2 and *RASSF1A*, with a significant increase in both mRNA and protein levels in MCF-7 and MDA-MB-231 cells. However, we did not observe significant regulation by lidocaine on viability of either cell line. Interestingly, lidocaine decreased the reduction in cell viability promoted by 0.2 μM cisplatin in both cell lines, with time- and dose-dependence. The sensitization effect of lidocaine on the anti-tumor effect of cisplatin was confirmed by clone assay; combined treatment with 0.1 mM lidocaine and 0.2 μM cisplatin significantly reduced the clone formation of MCF-7 cells compared to the singular 0.1 mM lidocaine treatment.

Taken together, lidocaine sensitizes the cytotoxicity of cisplatin against breast cancer cells. Cisplatin has been clinically utilized for breast cancer treatment for decades [37,38], and it mainly induces apoptotic death of breast cancer cells [41,43,55]. Cisplatin-induced apoptosis has shown to be sensitized by Mdm2 antagonists [56], by the expression of Siva-1 protein [57], or by theophylline [58]. Here, we demonstrate that lidocaine sensitized apoptosis promotion by cisplatin in MCF-7 cells. Though lidocaine did not directly induce apoptosis in MCF-7 cells, it enhanced cisplatin-induced apoptosis; treatments with both agents promoted significantly higher levels of apoptosis in MCF-7 cells. Western blot analysis confirmed sensitization by lidocaine. Treatment with both agents promoted higher expression or activation level of apoptosis-associated molecules, such as activated caspase 3, cleaved PARP and released cytochrome c. Moreover, apoptosis promotion by lidocaine and cisplatin treatment was inhibited by abrogation of the *RAR*β*2* or *RASSF1A* gene. Thus, we demonstrate here lidocaine sensitization to the cytotoxicity of cisplatin in MCF-7 breast cancer cells. However, further investigation is needed to clarify the exact mechanisms of sensitization to cisplatin-induced apoptosis in breast cancer cells through lidocaine-induced demethylation of *RAR*β2 and *RASSF1A* genes.

## 4. Materials and Methods

### 4.1. Cell Culture and Treatment with Reagents

MDA-MB-231 and MCF-7 breast cancer cell lines were provided by the cell resource center of the Chinese Academy of Medical Sciences (Beijing, China). MDA-MB-231 cells were grown in DMEM/F12 (HyClone, Logan, UT, USA), supplemented with 10% fetal bovine serum (FBS; Gibco, Rockville, MD, USA). MCF-7 breast cells were maintained in Dulbecco’s modified Eagle’s medium (Invitrogen, Carlsbad, CA, USA), supplemented with 10% FBS (Gibco, Rockville, MD, USA). Cells were cultured at 37 °C in 5% CO_2_ to 80%–90% confluence and were subjected to DMEM/F12 or DMEM supplemented with 2% FBS, containing 0.01, 0.02, 0.1, 0.5 or 1 mM lidocaine (Sigma–Aldrich, St. Louis, MO, USA), 10 μM 5-aza-2'-deoxycytidine (DAC) (Sigma–Aldrich) or (and) 0.2 μM cisplatin (Sigma–Aldrich for various hours for the DNA methylation sequencing, cell viability or cell apoptosis assay, and cell colony forming assay. DAC or cisplatin was utilized to as a positive demethylation agent or as an apoptosis inducer. To abrogate the expression of *RAR*β*2* or *RASSF1A*, siRNA–*RAR*β*2* (5'-CAGC UGAG UUGG ACGA UCU-3'), siRNA–*RASSF1A* (5'-GAC CUC UGU GGC GAC UUCA-3') or siRNA control (5'-AGCG AATT AGCT TGCC GTG-3') was synthesized by GenePharma Technology (Shanghai, China) and was transfected by lipofectamine 2000 (Thermo Fisher Scientific, Waltham, MA, USA) with a concentration of 50 nM according to the manufacurer’s guidance.

### 4.2. Methylation Analysis of Global Genomics and TSGs, RARβ2 and RASSF1A

To analyze the effects of 0.01, 0.1, or 1 mM lidocaine, with 10 mM DAC as positive demethylation agent, we extracted genomic DNA from MDA-MB-231 or MCF-7 cells post various treatment using DNeasy tissue kit (Qiagen, Hilden, Germany) according to the manufacturer’ manual. Sodium bisulfite conversion of genomic DNA in each sample was performed as described [59]. The DNA methylation was analyzed using MethyLight method [60], to quantify the methylation level of *RAR*β*2* and *RASSF1A*, both of which have reported to be hypermethylated in breast cancer [27,38]. The methylation status of individual CpG sites in each gene within the promoter region was determined by the sodium bisulfite-sequencing assay as described previously [61,62].

### 4.3. RNA Extraction and RT-qPCR

Total cellular mRNA samples were extracted from cultured cells using the RNeasy Mini Kit (Qiagen, Valencia, CA, USA). Quantification of *RAR*β*2* and *RASSF1A* expression was conducted using the QuantiTect SYBR Green PCR Kit (Qiagen, Hilden, Germany) in the LightCycle 2.0 (Roche, Mannheim, Germany). The sequences of the primers for *RAR*β*2* are as following, forward primer: 5'-TGAG TCCT GGGC AAAT CCTG-3', reverse primer: 5'-TTGA GAGC TTTC TCCT GGAG-3'. And the sequences of the primers for *RASSF1A* are as following, forward primer: 5'-AGCC TGAG CTCA TTGA GCTG-3', reverse primer: 5'-ACCA GCTG CCGT GTGG-3'. All mRNA expression levels were normalized to *β-actin* (forward primer: 5'-GATG AGAT TGGC ATGG CTTT-3', revserse primer: 5'-GTCA CCTT CACC GTTC CAGT-3'), and ^∆∆^*C*_t_ method was used for relative quantification [63].

### 4.4. Protein Isolation and Western Blot Analysis

Total cellular protein samples were prepared with a cell lysis reagent (Sigma–Aldrich) according to the manual and supplemented with a protease inhibitor cocktail (Pierce, Rockford, IL, USA). For the Cyt C release assay, the cytoplasmic protein was isolated by the Mitochondria/Cytosol Fractionation Kit (Abcam, Cambridge, UK). Each protein sample was separated by 10% SDS-PAGE gel and was transferred to a nitrocellulose membrane. Then the rabbit polyclonal antibody to *RAR*β*2* (Santa Cruz Biotechnology, Santa Cruz, CA, USA), *RASSF1A* (Abcam, Cambridge, UK), caspase 3 (Sino Biological, Beijing, China), cleaved-PARP (Cell Signaling Technology Inc., Danvers, MA, USA), Cytochrome c (Santa Cruz Biotechnology, Santa Cruz, CA, USA) or *β-actin* (Sino Biological, Beijing, China) was used to detect the protein level of each molecule. Goat anti-rabbit IgG conjugated to horseradish peroxidase (Pierce) and ECL detection systems (Super Signal West Femto; Pierce) were used for detection.

### 4.5. MTT Assay and Cell Colony Formation Assay

Cell viability was determined by MTT cell viability assay kit (Biotium Inc., Beijing, China). MCF-7 or MDA-MB-231 cells were seeded in 96-well plates and were incubated at 37 °C for 24 h to approximately 85% confluence, and then cells were treated with 0.1 mM lidocaine, 0.2 μM cisplatin or with both agents for various hours. Then cells were incubated with 10 μL MTT and were incubated at 37 °C for 4 h. Post addition of 200 μL DMSO into each well to dissolve the formazan, the absorbance was measured on an ELISA plate reader with a test wavelength of 570 nm and a reference wavelength of 630 nm to obtain sample signal (OD_570_–OD_630_). For cell colony formation assay, 4 × 10^2^ cells were incubated in 6-well plates at 37 °C containing 5% CO_2_, and were treated with 0.1 mM lidocaine, 0.2 μM cisplatin or with both agents. 96 h post treatment, cells were stained with crystal violet (0.005%) for 30 min and colony numbers were counted.

### 4.6. Cell Apoptosis Assay and Fluorometric Analysis of Caspase 3 Activity

Apoptosis of MCF-7 or MDA-MB-231 cells was examined with an annexin V/FITC apoptosis detection kit (Abcam, Cambridge, UK). Briefly, approximate 6 × 10^5^ cells post treatment were stained with annexin V-FITC and propidium iodide and detected by a FACScan flow cytometer (BD Biosciences, San Jose, CA, USA). The apoptosis was evaluated by a percentage of apoptotic cells to total cells. The caspase 3 activity was examined with an AMC Caspase Profiling Kit (for caspase 3) (AnaSpec, Fremont, CA, USA) according to the manual. MCF-7 cells (105 cells) post treatment were collected, and the cell pellets were washed with phosphate-buffered saline, then caspase-3-like activity was determined by assessment of Asp-Glu-Val-Asp (DEVD)-AMC cleavage. Briefly, pellets were transferred to a microtiter plate and were resuspended in 100 μL (final volume) of a caspase buffer solution supplemented with the fluorogenic peptide substrate Ac-DEVD-AMC. The cleavage was monitored over a 30 min period at 37 °C in a Fluoroscan II plate reader using an excitation wavelength of 390 nm and an emission wavelength of 460 nm. And the activity was expressed as a relative value of fluorescence intensity of AMC to control.

## 5. Conclusions

In conclusion, we have confirmed the DNA demethylation effects of lidocaine in breast cancer cells, and demonstrated the lidocaine-sensitized cytotoxicity of cisplatin against MCF-7 cells via enhancement of cisplatin-induced apoptosis.

## Supplementary Information

The supplementary figures can be found at http://www.mdpi.com/1422-0067/15/12/23519/s1.
